# Electroceuticals for Neurogastroenterology and Motility Disorders

**DOI:** 10.1007/s11894-023-00866-9

**Published:** 2023-03-03

**Authors:** Yan Jiang, Edy Soffer

**Affiliations:** Division of Gastrointestinal and Liver Diseases, Department of Internal Medicine, University of Southern California, 1520 San Pablo Street, Los Angeles, CA 90033 USA

**Keywords:** Electrostimulation, Gastroparesis, Vagal nerve stimulation, Sacral nerve stimulation

## Abstract

**Purpose of Review:**

To provide an updated overview on use of electrostimulation in gastrointestinal motility disorders and obesity, with a focus on gastric electrical stimulation, vagal nerve stimulation and sacral nerve stimulation.

**Recent Findings:**

Recent studies on gastric electrical stimulation for chronic vomiting showed a decrease in frequency of vomiting, but without significant improvement in quality of life. Percutaneous vagal nerve stimulation shows some promise for both symptoms of gastroparesis and IBS. Sacral nerve stimulation does not appear effective for constipation. Studies of electroceuticals for treatment of obesity have quite varied results with less clinical penetrance of the technology.

**Summary:**

Results of studies on the efficacy of electroceuticals have been variable depending on pathology but this area remains promising. Improved mechanistic understanding, technology and more controlled trials will be helpful to establish a clearer role for electrostimulation in treatment of various GI disorders.

## Introduction

The gastrointestinal (GI) tract has extensive interactions with our body’s central nervous system through the vagus, thoracolumbar and sacral nerves [1••]. Innervation of the enteric nervous system can produce a variety of end actions such as peristalsis, hormone secretion and inflammatory modulation [1••]. As such, electrostimulation has been touted as an alternative to pharmacotherapy for treatment of various gastrointestinal diseases [2].

Varying electrostimulation parameters have been used for treatment and functionality can in general be classified functionally as excitatory or inhibitory [3]. Some have classified different configurations of stimulation as long-pulse width or short-pulse width (as well as use of trains/intermittent short pulses) [3]. Long-pulse was the initial method used for pacing and only these long pulse widths (tens to hundreds of ms) electrostimulation can alter and activate muscle function. Short-pulse widths (in order of hundreds of μs), which are the current stimulation parameter used in gastric stimulation, do not affect muscle function, but rather seem to activate nerve fibers of the autonomic and enteric nervous system.

Despite the enthusiasm around these therapies, results of studies on the efficacy of electroceuticals for treatment of GI disorders have been variable. In this review, we will highlight the latest advances in technology and application of electroceuticals in neurogastrointestinal and motility disorders as well as obesity, with a particular focus on gastric electrical stimulation (GES), vagal nerve stimulation (VNS), sacral nerve stimulation (SNS).

## Gastric Electrical Stimulation

**Fig. 1:**
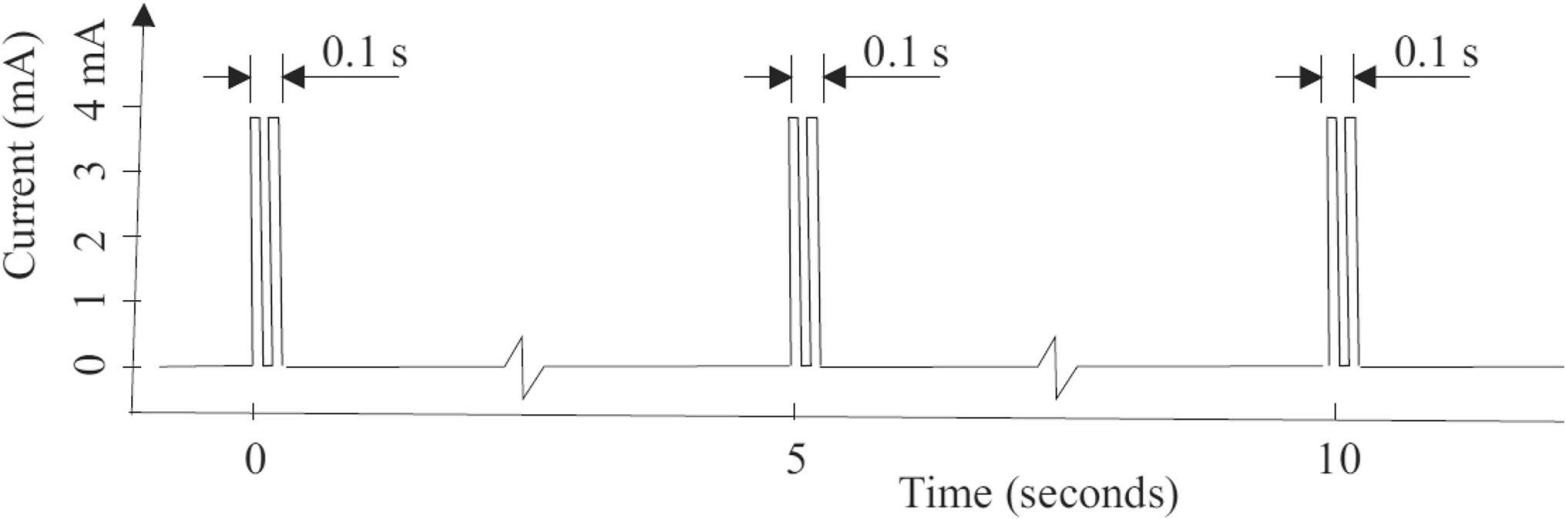
An illustration of the type of electrical stimulation delivered by the Enterra system for gastroparesis. Short bursts of short duration rectangular pulses (330 μsec each) are given at a frequency of 14 Hz in each burst. Bursts last 0.1 s, and are delivered every 5 s. This is the default setting, but variables can be adjusted depending on clinical response. This type of stimulus is referred to as short duration/high frequency stimulation, and also as low energy stimulation. (reproduced from: *Soffer E. Gastric Electrical Stimulation for Gastroparesis. Journal of Neurogastroenterology and Motility 201;18:131–137*)

Currently, GES (Enterra™, Medtronic) can be considered a treatment option for refractory vomiting, often in the setting of gastroparesis, but this is not a definite requirement. Placement of Enterra involves surgical placement of two leads, 1 cm apart, on the greater curvature of the stomach, 10 cm away from the pylorus. These leads are connected to a stimulator placed subcutaneously in the abdominal wall. The device provides low energy stimulation that allows low power consumption that extends battery life (Fig. 1). The precise mechanisms responsible for the beneficial effect of GES are not altogether clear. The current system in clinical use (Enterra) delivers trains of short pulses (330 μs), at high frequency (14 Hz for 0.1 s, repeated every 5 s) and of low energy (in the range of 5 mA) [4]. These pulse parameters are not suitable for gastric pacing and do not have an impactful effect on gastric emptying. Current evidence suggests that electrical stimulation activates afferent pathways to the brain, as well as brain activity, which points to possible activation of central control mechanisms for nausea and vomiting [5, 6]. A neurohumoral mechanism may involve release of ghrelin, a hormone associated increased appetite and antinausea effect, from neuroendocrine cells in the stomach [7, 8].

Clinical guidelines state that GES can be considered for control of gastroparesis symptoms as a humanitarian use device (as approved by FDA, conditional recommendation, low quality of evidence) [9]. To date, there have been mixed results from trials of GES for refractory vomiting [10]. An initial double blinded, randomized, placebo-controlled trial reported reduction in vomiting frequency as well as symptomatic improvement [11]. However, subsequent trials in patients with gastroparesis did not show similar positive results, in part due to variation in sample size and design of the on and off crossover periods [4, 12]. Safety profiles have been reported in a systematic review, with an adverse event rate of 8.7% in the immediate post operative period [10]. Repeat operations were done in about 11.1% and removal of GES device in 8.4% (Table 1).

**Table 1:** RCTs for gastric electrical stimulation

Study	Indication	Design	*N*	Results
Abell et al. [11]	Gastroparesis	Double blinded, crossover RCT of GES for 1 month on vs 1 month off. Then, prospective monitoring of GES on for 12 months	33	Self reported vomiting decreased in the on period compared to off (*p* < 0.05). In unblinded portion, symptoms improved at 12 months compared to baseline
McCallum et al. [4]	Diabetic gastroparesis	Double blinded, crossover RCT of GES for 3 months on vs 3 months off after an initial 6 week post implantation on period. Then, prospective monitoring of GES on for 4.5 months	55	Initial 6 weeks of GES on for all patients showed decrease in vomiting frequency. However, there was no difference in vomiting frequency during 3 month crossover period for GES on vs off (*p* = 0.215)
McCallum et al. [12]	Idiopathic gastroparesis	Double blinded, crossover RCT of GES for 3 months on vs 3 months off after an initial 6 week post implantation on period. Then, prospective monitoring of GES on for 4.5 months	32	Initial unblinded 6 weeks of GES on for all patients showed decrease in vomiting frequency. There was a slight, but not significant, reduction in vomiting frequency during 3 month crossover period for GES on vs off (*p* > 0.10)
Ducrotte et al. [13••]	Refractory vomiting	Double blinded, crossover RCT of GES for 4 months on and 4 months off	172 (133 with gastroparesis)	Improved vomiting symptoms during the on period compared to off (*p* < 0.001), regardless of baseline gastric emptying status. Quality of life scores were not improved with GES on

More recently, a multicenter, randomized, double-blinded crossover study was conducted to evaluate the efficacy of Enterra in patients with refractory vomiting, with or without gastroparesis [13••]. The study evaluated 172 patients (133 with gastroparesis) for 4 months after implantation of GES device and were randomized to having the device on vs off; 149 patients then crossed over to the other group for 4 months. Patients in the device on group experienced less vomiting frequency than the control (*p* < 0.001), in both phases of the crossover trial. This was the case regardless of diabetes status and irrespective of gastric emptying time. However, despite the reported reduction in vomiting frequency, having the GES on was not associated with an increased quality of life. A substantial placebo effect was observed in the sham stimulated group.

In the open label follow up of the aforementioned trial, an additional 2 year follow up was conducted [14]. Quality of life scores increased during this time period, and more so in the non-diabetic population (*p* < 0.001). A cost-effective analysis was conducted and considering hospitalizations, time off work, transportation, etc., Enterra therapy was reported to save about $3,348 US dollars per patient/ per year. Though 25.4% of patients had one device related adverse event during the follow up period, no major complications leading to device removal were observed. Another recent study retrospectively examined the long term impact of GES over a 10 year period [15]. A little over half (54%) of patients reported improvement from baseline in symptoms other than epigastric pain. This was associated with an increase in quality of life survey scores from baseline (*p* = 0.005). However, the lack of well validated predictors of success of GES remains a limiting factor in the wider application of this therapeutic modality.

Optimization of pulse parameters and energy delivery can be attempted for patients who do not respond to GES using initial parameters, or whose symptoms worsen despite therapy. One algorithm applied an increased current, followed by an increase in ON time delivery if symptoms did not improve. Finally, one could also increase frequency as well, but with steps separated by a few months from each other [16]. While variations of such steps are commonly used, supporting evidence remains limited. All the above changes consume more power and tend to shorten battery life, below the expected 7–10 years when using default parameters.

## Vagal Nerve Stimulation

Vagal nerve stimulation is involved in the modulation of multiple processes and its impact in GI disorders such as gastroparesis and irritable bowel syndrome (IBS) is thought to be through regulation of central mechanisms of nausea/vomiting and alteration of visceral hypersensitivity [2, 17–19]. FDA approved devices for VNS have typically been used for epilepsy and depression but require surgical implantation. More recently, a handheld transcutaneous non-invasive VNS device (gammaCore™, electroCore) has been approved for treatment of headaches [20]. It is applied directly to the neck and targets the cervical branch of the vagus nerve [21].

For treatment of gastroparesis, the gammaCore VNS device has been studied in two small open label trials. An initial study of 35 patients with gastroparesis refractory to pharmacotherapy analyzed the 23 patients who were compliant with study procedures [22]. Participants were asked to do 2-min stimulations 12 times per day in 3 separate sessions (2 stimulations on each side of the neck). During the third week of treatment, this was increased to 18 times per day over 3 sessions (3 stimulations on each side of the neck). Response was classified as a decrease in the Gastroparesis Cardinal Symptom Index (GCSI) of at least 1 (0 = no symptoms, 5 = very severe symptoms). After 3 weeks, 8/23 (35%) patients were classified as responders and after 6 weeks, 10 total patients (43%) responded. No serious adverse events were reported, with only 1 case of skin irritation and 1 case of neck discomfort.

A more recent study was conducted with gammaCore in 15 patients with idiopathic gastroparesis [23•]. Cervical application was delivered for at least 4 weeks and consisted of 2 stimulations on each side of the neck, twice daily. There was symptom improvement during treatment course with 6/15 (40%) meeting primary endpoint of at least a 0.75 decrease in the composite GCSI. Though there was some suggestion of a reduction in gastric emptying time with treatment of VNS (T1/2 155 vs 129 min, CI -0.4 to 45), there was no difference in improvement of emptying times between responders vs non-responders. Treatment was safe and no adverse events were reported.

In addition to gastroparesis, percutaneous electrostimulation has been studied for treatment of irritable bowel syndrome. This has been mostly done with the Neuro-Stim device (now IB-Stim™, Innovative Health Solutions) via auricular stimulation of multiple cranial nerves including the vagus nerve. The device consists of a battery powered generator (placed behind ear) and four electrode wires which are placed on the outer ear. As previously mentioned, the proposed mechanism is improvement of visceral hypersensitivity. Recently, an analysis of adolescents with IBS who were treated with Neuro-Stim was done [24]. Data extracted from the research group’s prospective study from 2015–2016 of adolescents with functional abdominal pain disorders for auricular neurostimulation [25]. A total of 50 IBS patients were included for analysis, with 23 receiving sham and 27 receiving active therapy. Treatment was provided for 4 weeks and consisted of the device being on each week for 5 days with 2 days off. The primary endpoint was the number of patients with a reduction of at least 30% of the worst abdominal pain severity and this was met in 59% of active therapy participants vs 26% of patients who received sham stimulation (*p* = 0.024). Patients who received active therapy also had a lower median composite pain score than those who received sham stimulation. No significant adverse events were reported.

A more recent study of transauricular vagal nerve stimulation was done on 42 adults with constipation predominant IBS [26]. This is a different device than the Neuro-Stim as one pair of electrodes is placed at the bilateral concha (SNM-FDC01, Ningbo Maida Medical Device Inc). After four weeks of treatment, there was improvement in visual analog pain, quality of life and IBS symptom scores in those who received active treatment compared to sham stimulation (all *p* < 0.05). There was also an increase in the number of spontaneous bowel movements per week (2.8 vs 0.9, *p* = 0.001).

## Sacral Nerve Stimulation

SNS (InterStim™, Medtronic), when applied to GI disorders, is applied mainly toward treatment of fecal incontinence and constipation [27, 28]. The procedure involves surgical placement of an electrode lead system at the sacral foramen, which is attached to a pulse generator implanted in the subcutaneous tissue of the buttocks. Prior to implantation of a more permanent stimulator, this system allows for a trial of SNS with a temporary percutaneous device first. The mechanism of action of SNS is not completely understood but is thought to be through its effects on afferent nerve activity [27]. For example in fecal incontinence, it has been hypothesized that the activation of these neural pathways causes reflex inhibition of sphincter function and increases rectal contractility [27, 29].

SNS for fecal incontinence dates back to the mid 1990s [30]. A Cochrane review was conducted in 2015 assessing the studies of SNS on fecal incontinence [28]. Six trials for fecal incontinence were included, 4 were cross over studies comparing on and off SNS and 2 were parallel groups comparing SNS to medical therapy and SNS to percutaneous tibial nerve stimulation (PTNS) [31–36]. In a trial of 40 patients comparing SNS to PTNS, 61% had at least a 50% reduction in fecal incontinence episodes at 6 months compared to 47% in the PTNS group [36]. A prior study in patients with severe fecal incontinence compared SNS to optimal medical therapy (pelvic floor exercises, bulking agents, etc.) and found that those who had SNS had significantly less episodes of incontinence at both 3 and 12 months [33]. There was also improved quality of life scores (in contrast to no improvement in the medical therapy group) as well as a reported 47.2% of patients with SNS achieving perfect continence. In 3 of 4 crossover trials of SNS, there seemed to be a benefit of SNS with less reported fecal incontinence episodes during periods of on stimulation compared to off periods [31–35]. Though only some studies reported adverse events, these were generally low and included pain at stimulator site, hematoma formation, and infection—some of which resulted in removal of the device. In those who have failed conservative therapy, SNS can be an option to improve fecal continence.

The data of SNS for constipation is less promising. Although some initial non-controlled data had been promising, more recent randomized, controlled trials have not demonstrated similar efficacy [37, 38]. In a study of 55 patients who received permanent SNS implantation after 3 weeks of temporary peripheral nerve stimulation, the proportion of patients who reported a bowel movement with feeling of complete evacuation on at least 2 days of the week for 2 of 3 weeks (primary outcome) did not differ between active and sham stimulations [37]. A more recent trial demonstrated similar results [38]. Those who demonstrated response to temporary peripheral nerve stimulation for 3 weeks were offered a permanent stimulator and included in the trial consisting of two randomly assigned 8 week intervals of active or sham stimulation (20/36 patients). Positive response to therapy was defined as at least 3 bowel movements per week and/or more than 50% in improvement of symptoms. There was no difference in the response rates after active and sham periods (12/20 vs 11/20, *p* = 0.746).

From a practical standpoint, the standard SNS parameters for frequency and pulse duration are 14 Hz and 210 µs, respectively. but the effects of changing parameters of stimulation are unclear [39, 40]. A small study of 12 patients looked at the effect of changing these pulse parameters (low or high frequency vs low or high pulse duration) on fecal incontinence [40]. Of the 8 patients who experienced improvement in symptoms, 6 had increased frequency (31 Hz, 210 µs) while 2 had low pulse width (14 Hz, 90 µs). Settings can be changed during both the testing and chronic stimulator phases and there can be preset parameters customized for patients [39].

## Obesity

Electroceuticals for weight loss have garnered lots of interest given the rising healthcare issues and costs related to obesity and lack of effective long term pharmacotherapeutic options [41]. Electrostimulation for treatment of obesity have typically been either with direct gastric placement or via modulation of the vagus nerve. Signals for hunger and satiety are thought to be communicated through the vagal afferents and vagotomy has been shown to result in weight loss [1••, 42, 43].

The vBloc Therapy Maestro® (EnteroMedics) applies this concept by producing a blocking electrical stimulation to both the anterior and posterior vagus nerves at the gastroesophageal junction. It is FDA approved for the treatment of obesity. The ReCharge trial was a randomized double blinded sham controlled study evaluating the effect of vagal nerve blockade on morbid obesity [44]. It was designed to address limitations of the EMPOWER study which did not show significant differences in weight loss between treatment and control, but did show that those who received 12 h per day of vagal blockade achieved the anticipated weight loss results [45]. In the ReCharge trial, patients with BMI 40–45 or 35–40 with 1 or more obesity related comorbidity were enrolled with 162 receiving vBloc and 77 getting sham device. At 12 months, excess weight loss (EWL) was 24.4% in the vBloc group compared to 15.9% in the sham control (95% CI 3.1–13.9). This, though statistically significant, did not meet the 10 point margin of difference prespecified as a co-primary endpoint. In addition, at 12 months, 52% of patients in the vBloc group achieved 20% or more EWL and 38% achieved 25% or more EWL. This also did not meet the co-primary endpoint of 55% of patients and 45% patients, respectively. The rate of serious adverse events related to the device, implantation or therapy was 3.7%. Follow up of participants at both 18 and 24 months showed sustained EWL of 23% and 21%, respectively [46, 47].

GES is another method that has been studied for treatment of obesity with varying results. It is thought to induce early satiety, but the exact mechanism is unknown. Electrodes are typically placed at the anterior wall, but later generations of GES devices also had a closed loop feedback system with electrodes in the fundus to detect gastric distention and adjust GES accordingly [1••]. Though there does not appear to be much clinical penetrance, a few GES systems have been tested for treatment of obesity. The SHAPE trial tested the Transcend™ system (Medtronic) [48]. This double blinded, randomized, placebo-controlled trial enrolled 190 patients who received the gastric stimulator and were randomized to either on or off stimulation. There was no difference in EWL between the treatment and control groups (11.8% vs 11.7%, *p* = 0.717). A study of 34 patients implanted with the abiliti® closed loop system (IntraPace) did show 28.7% EWL at 12 months and 27.5% at 27 months [49]. However, this study did not have any control arms, which limits the interpretability of actual effectiveness of this system. Similarly, the Diamond system (formerly Tantalus, MetaCure) had been shown to decrease weight in a small, open label, non-controlled study [50]. More recently, this system was studied in patients with type 2 diabetes and though weight loss was not the purpose or main outcome of the study, there did not appear to be a significant difference in weight change [51]. To date, use of electrical stimulation for weight loss has not fulfilled initial expectations.

## Conclusions

More recent studies of GES for gastroparesis seem to show some effectiveness for chronic vomiting symptoms, but may not improve quality of life. Initial studies of percutaneous VNS demonstrate promise for both gastroparesis and IBS but controlled trials are necessary. SNS has been used for fecal incontinence with some success but does not appear helpful for chronic constipation. Electrostimulation for treatment of obesity have had varying results with limited randomized controlled trials to demonstrate clear efficacy. Use of electroceuticals in various gastrointestinal disorders remains a promising area of research, especially as our technological and pathophysiological understanding of these complex GI disorders continue to improve. However, robust trials, with adequate design, using sham stimulation, are essential in proving the effect of electrical stimulation in the GI tract.
